# The Apoptotic Effect of Caffeic or Chlorogenic Acid on the C32 Cells That Have Simultaneously Been Exposed to a Static Magnetic Field

**DOI:** 10.3390/ijms23073859

**Published:** 2022-03-31

**Authors:** Magdalena Kimsa-Dudek, Agnieszka Synowiec-Wojtarowicz, Agata Krawczyk, Agnieszka Kosowska, Małgorzata Kimsa-Furdzik, Tomasz Francuz

**Affiliations:** 1 Department of Nutrigenomics and Bromatology, Faculty of Pharmaceutical Sciences in Sosnowiec, Medical University of Silesia, Katowice, Jednosci 8, 41-200 Sosnowiec, Poland; asynowiec@sum.edu.pl (A.S.-W.); akrawczyk@sum.edu.pl (A.K.); 2 Department of Biochemistry, Faculty of Medical Sciences in Katowice, Medical University of Silesia, Medyków 18, 40-752 Katowice, Poland; akosowska@sum.edu.pl (A.K.); malgorzata.kimsa@sum.edu.pl (M.K.-F.); tfrancuz@sum.edu.pl (T.F.)

**Keywords:** apoptosis, polyphenols, static magnetic field, cancer cells, chlorogenic acid, caffeic acid

## Abstract

The induction of apoptosis is one of the main goals of the designed anti-cancer therapies. In recent years, increased attention has been paid to the physical factors such as magnetic fields and to the natural bioactive compounds and the possibilities using them in medicine. Hence, the aim of this study was to evaluate the anti-tumor effect of caffeic or chlorogenic acid in combination with a moderate-strength static magnetic field on C32 melanoma cells by assessing the effect of both factors on the apoptotic process. The apoptosis of the C32 cells was evaluated using a flow cytometry analysis. The expression of the apoptosis-associated genes was determined using the RT-qPCR technique. The caspase activity and the concentration of the oxidative damage markers were also measured. It was found that phenolic acids and a static magnetic field trigger the apoptosis of the C32 cells and also affect the expression of the genes encoding the apoptosis regulatory proteins. In conclusion, our study indicated that both of the phenolic acids and a static magnetic field can be used supportively in the treatment of melanoma and that caffeic acid is more pro-apoptotic than chlorogenic acid.

## 1. Introduction

Apoptosis is a physiological process whose role is to remove abnormal or damaged cells, which is essential for maintaining tissue homeostasis. The main regulators of apoptosis include both the pro-survival and pro-apoptotic proteins of the Bcl-2 (B-cell CLL/lymphoma 2) family such as Bcl-2 (B-cell lymphoma 2), Bcl-x (BCL2 like 1) with Bcl-xL (B-cell lymphoma extra-large) and Bax (Bcl-2-associated X protein) as well as the caspases. Caspases are a crucial element in the apoptotic signal cascade. Taking into account their role in mammalian cells, they can be divided into the following groups: initiator (2, 8, 9 and 10); effector (3, 6 and 7); or inflammatory (1, 4, 5, 11 and 12) [1]. Programmed cell death can be triggered by various external factors or by the internal condition of cells. Disturbances in the course of this process are observed in many pathological states such as cancer diseases [2]. An example is melanoma, which is characterized by a high level of resistance to chemotherapeutic drugs. Furthermore, the treatment of a relatively rare type of this cancer, amelanotic melanoma, which is usually diagnosed at an advanced stage, poses a great therapeutic challenge. The difficulties in treating melanoma result from mechanisms of resistance that cancer cells have developed against programmed death and changes in the course of apoptosis at the molecular level, including the activation of the anti-apoptotic factors and the suppression of the pro-apoptotic factors or the enhancement of the survival signals [3]. Therefore, the induction of the apoptosis process is one of the main goals of the designed anti-cancer therapies [4].

Scientific research is increasingly focused on the search for effective new therapies that are based on bioactive compounds from natural sources. Polyphenols belong to the plant secondary metabolites that are found in edible plant products and medicinal plants. Phenolic acids constitute an important group of polyphenolic compounds and one of their most significant properties is their antioxidant activity, whereby this activity is more pronounced in the case of the derivatives of hydroxycinnamic acid such as caffeic (CA) or chlorogenic acid (CGA, an ester of caffeic acid and quinic acid) [5,6], which are present at high levels in many foods, including coffee. They are the bioactive components of a diet that also influence the intracellular signaling cascades. Much research has shown that dietary polyphenols might have chemopreventive effects on various cancers through different mechanisms, including inhibiting DNA synthesis, modulating the production of reactive oxygen species (ROS), regulating the cell cycle and proliferation. Moreover, polyphenols can also modulate the expression of the apoptotic regulatory proteins and thus can affect programmed cell death. Their pro-oxidative properties (depending on the concentration) are also observed via stimulating the formation of oxygen free radicals [7,8].

In recent years, increased attention has been paid to the physical factors such as magnetic fields and the possibilities of their use in medicine. Many studies have concerned the influence of an SMF (static magnetic field) on the various biological processes that are important in the pathogenesis and treatment of different diseases. It has been reported that a moderate or strong intensity SMF can inhibit cancer cell growth [9,10,11]. In turn, the studies of Chen et al. [12] suggested its synergistic antitumor effect together with a naturally derived compound, capsaicin, on liver cancer cells (HepG2).

Therefore, it seems advisable to consider the physical factors, such as an SMF and the bioactive compounds that are present in food, as potential agents for supporting anti-cancer therapy. Hence, the aim of this study was to evaluate the anti-tumor effect of caffeic or chlorogenic acid in combination with a moderate-strength static magnetic field on C32 melanoma cells by assessing the effect of both factors on the apoptotic process.

## 2. Results

### 2.1. Effect of Chlorogenic or Caffeic Acid at a Concentrations of 1 mmol/L and a Static Magnetic Field with A 0.7 T Flux Density on the Apoptosis Process

The analysis of the apoptosis process of the C32 cells that had been treated with chlorogenic or caffeic acid and exposed to an SMF was performed using a flow cytometer.

**Figure 1:**
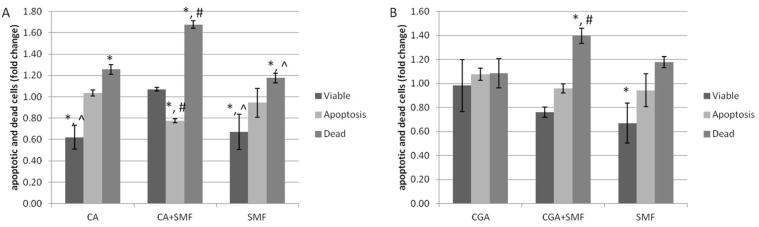
Apoptosis of the C32 cells after exposure to the phenolic acids at a concentration of 1 mmol/L and a static magnetic field with a 0.7 T flux density (**A**—caffeic acid, **B**—chlorogenic acid). The results are presented as fold changes relative to the control cells and are reported as the mean ± SD, statistical significance: * *p* < 0.05 vs. C, # *p* < 0.05 vs. CA/CGA, ^ *p* < 0.05 vs. CA/CGA + SMF, C—control cultures, CA/CGA—caffeic or chlorogenic acid-treated the C32 cells, CA/CGA + SMF—cells that had simultaneously been exposed to phenolic acid and a static magnetic field, SMF—cells that had only been exposed to a static magnetic field.

It was observed that the caffeic acid increased the number of apoptotic and dead cells and reduced the number of viable cells (Tukey post hoc test, *p* < 0.05). The number of dead cells also increased as a result of exposure to an SMF and both factors (Tukey post hoc test, *p* < 0.05) (Figure 1A). Similar results were noted for cancer cells after they were exposed to chlorogenic acid, an SMF or both (Figure 1B).

It was also revealed that caffeic acid caused a statistically significant reduction in the number of viable cells compared to the cells that had been cultured in the presence of chlorogenic acid (Tukey post hoc test, *p* = 0.006). On the other hand, the difference in the number of apoptotic and dead cells between the CA- and CGA-treated C32 cells was not statistically significant. Our results also showed a statistically significant increase in the number of dead cells after the simultaneous exposure to CA and an SMF compared to the cells exposed to CGA and an SMF (Tukey post hoc test, *p* < 0.001).

Based on the analysis of the CDI value (coefficient of drug interaction), it can be seen that both of the phenolic acids combined with an SMF exhibited a synergistic apoptotic effect on the C32 cells (CDI = 0.541 ± 0.211 for CA and CDI = 0.546 ± 0.102 for CGA).

### 2.2. Effect of Chlorogenic or Caffeic Acid at a Concentration of 1 mmol/L and a Static Magnetic Field with A 0.7 T Flux Density on the Expression of the Apoptosis-Related Genes and Proteins

The analysis of the relative mRNA expression of the genes related with apoptosis in the C32 cells after exposure to CA/CGA and a moderate SMF was analyzed using the RT-qPCR technique.

**Figure 2:**
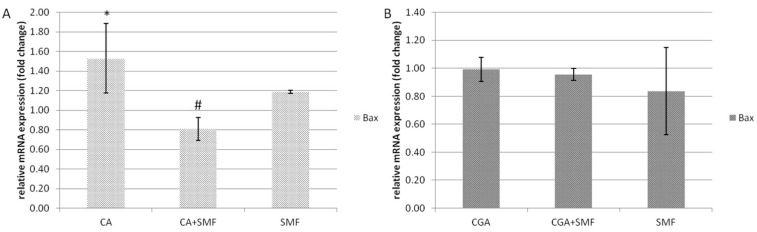
The relative mRNA expression of the *Bax* gene in the C32 cells after exposure to the phenolic acids at a concentration of 1 mmol/L and a static magnetic field with a 0.7 T flux density (**A**—caffeic acid, **B**—chlorogenic acid). The results are presented as fold changes relative to the control cells and are reported as the mean ± SD, statistical significance: * *p* < 0.05 vs. C, # *p* < 0.05 vs. CA/CGA, C—control cultures, CA/CGA—caffeic or chlorogenic acid-treated the C32 cells, CA/CGA + SMF—cells that had simultaneously been exposed to phenolic acid and a static magnetic field, SMF—cells that had been exposed to a static magnetic field.

The relative mRNA expression of the pro-apoptotic *Bax* gene in the C32 cells after exposure to caffeic acid was statistically significantly higher by about 53.3% compared to the control cells (Tukey post hoc test, *p* = 0.043). However, in the CA and SMF-treated cells there was a reduction in the *Bax* gene expression compared to the CA-treated cells (Tukey post hoc test, *p* = 0.016). Moreover, a static magnetic field alone had no statistically significant effect on the relative mRNA expression of this gene (Figure 2A). In turn, in the cancer cells that had been treated with chlorogenic acid and an SMF, there were no significant differences in the mRNA level of the *Bax* gene (Figure 2B).

**Figure 3:**
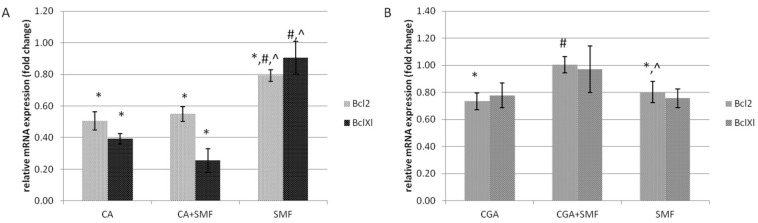
The relative mRNA expression of the *Bcl2* and *BclXl* genes in the C32 cells after exposure to the phenolic acids at a concentration of 1 mmol/L and a static magnetic field with a 0.7 T flux density (**A**—caffeic acid, **B**—chlorogenic acid). The results are presented as fold changes relative to the control cells and are reported as the mean ± SD, statistical significance: * *p* < 0.05 vs. C, # *p* < 0.05 vs. CA/CGA, ^ *p* < 0.05 vs. CA/CGA + SMF, C—control cultures, CA/CGA—caffeic or chlorogenic acid-treated the C32 cells, CA/CGA + SMF—cells that had simultaneously been exposed to phenolic acid and a static magnetic field, SMF—cells that had been exposed to a static magnetic field.

The expression of the anti-apoptotic *Bcl2* and *BclXl* genes was significantly down-regulated in the cells after exposure to caffeic acid and both caffeic acid and an SMF compared to the control cells (Tukey post hoc test, *p* < 0.001). In the C32 cells that had been cultured in the presence of chlorogenic acid, a similar effect was observed only for the *Bcl2* gene (Tukey post hoc test, *p* = 0.011). There was also a statistically significant decrease in the mRNA level of the *Bcl2* gene in the cells that had been exposed to the SMF alone, although only by approximately 20%. This indicates that an SMF alone had a much smaller effect on the expression of the anti-apoptotic genes (Figure 3A,B).

**Figure 4:**
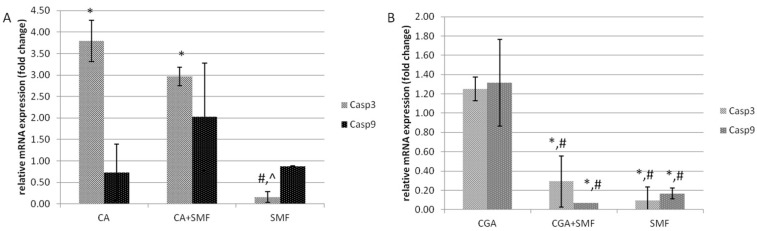
The relative mRNA expression of the *Casp3* and *Casp9* genes in the C32 cells after exposure to the phenolic acids at a concentration of 1 mmol/L and a static magnetic field with a 0.7 T flux density (**A**—caffeic acid, **B**—chlorogenic acid). The results are presented as fold changes relative to the control cells and are reported as the mean ± SD, statistical significance: * *p* < 0.05 vs. C, # *p* < 0.05 vs. CA/CGA, ^ *p* < 0.05 vs. CA/CGA + SMF, C—control cultures, CA/CGA—caffeic or chlorogenic acid-treated the C32 cells, CA/CGA + SMF—cells that had simultaneously been exposed to phenolic acid and a static magnetic field, SMF—cells that had been exposed to a static magnetic field.

In the case of caspase 3, the relative mRNA expression was higher after the cancer cells had been treated with the caffeic acid (3.5-fold) and both factors: chemical and physical (three-fold) (Tukey post hoc test, *p* = 0.008 and *p* = 0.043, respectively). Moreover, both factors did not have a statistically significant effect on the mRNA level of caspase 9. Furthermore, in the C32 cells that had been exposed to an SMF alone and an SMF combined with chlorogenic acid, there was a decrease in the relative mRNA expression of *Casp3* (Tukey post hoc test, *p* = 0.005 and *p* = 0.024, respectively) and *Casp9* (Tukey post hoc test, *p* = 0.027 and *p* = 0.021, respectively) (Figure 4A,B).

However, statistically significant differences in the mRNA level of the *Bax*, *Bcl2*, *BclXl*, *Casp3* and *Casp9* genes were observed between the CA- and CGA-treated cells (Tukey post hoc test, *p* < 0.001) and also between the CA + SMF- and CGA + SMF-exposed cells (Tukey post hoc test, *p* < 0.001). These differences suggest that caffeic acid has a stronger apoptotic effect on the C32 cells than chlorogenic acid.

**Figure 5:**
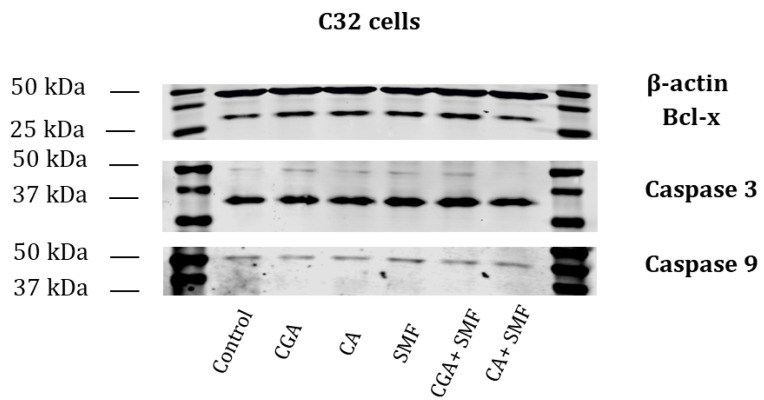
Western blot. B-actin, Bcl-x, Caspase 3, Caspase 9. Representative western blot image of the protein extracts from the C32 cells. Cell lines: control cells, CGA—chlorogenic acid-treated cells, CA—caffeic acid-treated cells, SMF—cell that had been exposed to a static magnetic field, CGA + SMF—cells that had simultaneously been exposed to chlorogenic acid and a static magnetic field, CA+—cells that had simultaneously been exposed to caffeic acid and a static magnetic field.

The presence of selected apoptotic proteins was also tested using the western blot. The presence of Bcl-x, caspase 3 and caspase 9 was observed in the C32 cells that had been treated with the phenolic acids and in the cells that had simultaneously been exposed to the phenolic acids and an SMF (Figure 5).

### 2.3. Effect of Chlorogenic or Caffeic Acid at a Concentration of 1 mmol/L and a Static Magnetic Field with a 0.7 T Flux Density on the Activity of the Caspases

As the caspases are the most important enzymes that are involved in the apoptosis process, the influence of both of the polyphenols and an SMF on the activity of the key initiator (caspase 9) and executive (caspase 3) caspases in the C32 cells was also investigated.

**Figure 6:**
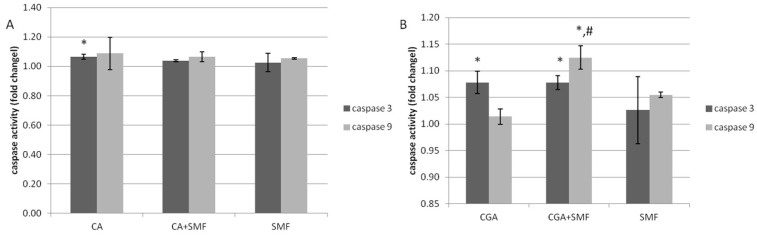
The caspase activity in the C32 cells after exposure to the phenolic acids at a concentration of 1 mmol/L and a static magnetic field with a 0.7 T flux density (**A**—caffeic acid, **B**—chlorogenic acid). The results are presented as fold changes relative to the control cells and are reported as the mean ± SD, statistical significance: * *p* < 0.05 vs. C, # *p* < 0.05 vs. CA/CGA, C—control cultures, CA/CGA—caffeic or chlorogenic acid-treated the C32 cells, CA/CGA+SMF—cells that had simultaneously been exposed to phenolic acid and a static magnetic field, SMF—cells that had been exposed to a static magnetic field.

The profile of the caspase activity results was similar to the results for the relative mRNA expression of the caspases. It was found that the activity of caspase 3 was statistically significant higher in the caffeic acid-treated cells compared to the control cells (Tukey post hoc test, *p* = 0.010). However, neither factor significantly affected the caspase 9 activity (Figure 6A). In the case of the cells that had been cultured with chlorogenic acid and simultaneously with CGA and an SMF, a statistically significant increase in the activity of caspase 3 was observed (Tukey post hoc test, *p* = 0.002 and *p* = 0.007, respectively). It was also revealed that both factors significantly affected the caspase 9 activity (Tukey post hoc test, *p* < 0.001) (Figure 6B).

Moreover, no statistically significant differences in the caspase 3 and caspase 9 activity were revealed between the CA- and CGA-treated cells, or with the cells that had simultaneously been exposed to an SMF.

### 2.4. Effect of Chlorogenic or Caffeic Acid at a Concentration of 1 mmol/L and a Static Magnetic Field with a 0.7 T Flux Density on the Concentration of the Oxidative Damage Markers

Apoptosis is closely related to oxidative stress, which is induced by the presence of an excessive amount of reactive oxygen species (ROS). ROS cause oxidative damage in the nucleic acids, proteins and lipids. Therefore, in the next stage of the study, the concentration of DNA/RNA oxidative damage markers was assessed.

**Figure 7:**
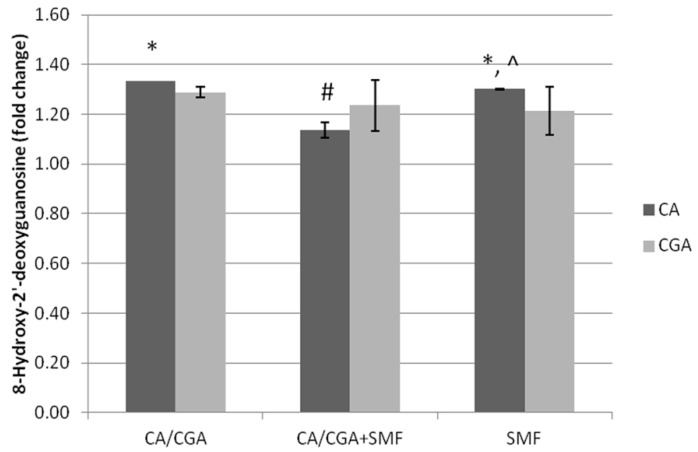
The DNA/RNA oxidative damage marker concentration in the C32 cells after exposure to the phenolic acids at a concentration of 1 mmol/L and a static magnetic field with a 0.7 T flux density. The results are presented as fold changes relative to the control cells and are reported as the mean ± SD, statistical significance: * *p* < 0.05 vs. C, # *p* < 0.05 vs. CA/CGA, ^ *p* < 0.05 vs. CA/CGA + SMF, C—control cultures, CA/CGA—caffeic or chlorogenic acid-treated the C32 cells, CA/CGA + SMF—cells that had simultaneously been exposed to phenolic acid and a static magnetic field, SMF—cells that had been exposed to a static magnetic field.

It was found that caffeic acid and an SMF caused a significant increase in 8-hydroxy-2’-deoxyguanosine concentration of 33.4% and 30.1% compared to the control cells (Tukey post hoc test, *p* = 0.003 and *p* = 0.004, respectively), whereas the combined action of both factors triggered a weaker effect (approximately 13.6%). The results also indicated that chlorogenic acid combined with an SMF did not significantly change the DNA/RNA oxidative damage marker concentration in cell culture medium (Figure 7).

We also did not observe significant changes in the concentration of oxidative damage marker between the groups of the C32 cells treated with CA and CGA, or after their simultaneous exposure to an SMF.

### 2.5. Effect of the Phenolic Acids at a Concentration of 1 mmol/L and an SMF with a 0.7 T Flux Density on the Intracellular ROS Production

Intracellular ROS production in the cells that had simultaneously been treated with the phenolic acids and an SMF was measured with flow cytometry using 2′,7′-dichlorodihydrofluorescein diacetate. Compared to the untreated cells, approximately 11% and 8% ROS, respectively, were generated in the cells that had been treated with CA and CGA. It was also observed that the exposure to an SMF alone caused an increase in the level of ROS (approximately 17%), whereas the combined action of both factors triggered a weaker effect than an SMF alone (Figures S1 and S2). We also did not observe any changes in the ROS level between the groups of the C32 cells that had been treated with CA and CGA or after their simultaneous exposure to an SMF.

## 3. Discussion

The anti-cancer properties of natural plant-derived antioxidants have been demonstrated in vitro in relation to various cell lines of breast, skin, colon, prostate, lung and bladder cancers and leukemia [13,14,15]. Acting as bioactive compounds, the polyphenols affect the process of the expression of many genes, and the molecular mechanism of their action is mainly associated with the redox homeostasis of cells, inflammation, the cell cycle and the apoptosis regulation and modulation of the essential cell signaling pathways [13].

Caffeic and chlorogenic acid are the main antioxidants that are found in coffee. Several studies have confirmed their biological effect on cancer cells. Villota et al. [16] showed their cytotoxicity activity and their ability to inhibit the migration process in the colorectal cancer cell lines: SW480 and SW620. The induction of apoptosis and the regulation of the cell cycle by compounds of coffee extracts have also been found in prostate cancer cells [17] and K562 chronic myeloid leukemia cells [18]. However, only a few studies concern the effect of phenolic acids on skin cancer or melanoma [19,20,21]. Our study indicated that both caffeic and chlorogenic acid at a concentration of 1 mmol/L are characterized by pro-apoptotic properties in relation to human amelanotic melanoma cells.

The SMF is a physical factor, the use of which in medicine and disease therapy has excited the increased interest of scientists. Few studies have shown the possibilities of its use in the treatment of cancer. Zafari et al. [11] investigated the effect of a moderate-strength SMF on the proliferative activity of human cervical cancer cells (HeLa cell line). They also suggested that the exposure of HeLa cells to an SMF might induce the lipid peroxidation process due to the observed increase in the malondialdehyde concentration. However, the molecular mechanisms of an SMF action on cells still remains unclear. Importantly, we found no studies on the combined effects of phenolic acids and an SMF on cancer cells. Hence, the aim of our research was to evaluate the common interaction of phenolic acids and an SMF that was generated by permanent magnets on melanoma cells.

Melanoma, especially in its late stage, is extremely difficult to treat, which correlates with its high mortality rate. Therefore, new strategies are being sought to support the current treatments. In addition, the use of natural compounds might reduce the side effects of standard therapy. Previous studies have shown that polyphenols demonstrate anticancer properties on melanoma cell lines via their cytotoxic and anti-proliferative effects and also by participating in the regulation of cell cycle, apoptosis and autophagy [22]. Martino et al. [23] suggested that the extract from *Larrea divaricata* Cav. could be an adjunctive therapy for melanoma due to its high content of polyphenols and their inhibitory effect on the proliferation of B6F10 cells via the inhibition of STAT3 and the induction of apoptosis. In turn, Huang et al. [24] showed similar properties of rosmarinic acid on melanoma A375 cells. They also found that this compound increases the sensitivity of cancer cells to cisplatin. Additionally, in our study, we indicated that polyphenols–caffeic and chlorogenic acid might have the potential to induce the apoptosis process in human melanoma C32 cells. It should also be taken into account that the oxidative stress that is generated by reactive oxygen species is closely associated with the induction of apoptosis. Previous studies have shown that these phenolic acids can disrupt redox homeostasis in the C32 cells, which can then trigger apoptosis [25,26]. Hence, high concentrations of polyphenols might exhibit pro-oxidative properties that could be used in the adjunctive treatment of cancer [13]. This was also confirmed by the results that showed an increase in the intracellular ROS level and in the concentration of the oxidative damage marker as a result of the exposure of the cells to both of the tested factors compared to the control cells.

Proteins from the Bcl-2 family are important regulators of apoptosis, and therefore in our study we determined the expression of selected proteins from this family in the melanoma cells after they had been exposed to the phenolic acids and an SMF. The increase in the expression of pro-apoptotic *Bax* and the decrease in the mRNA level of anti-apoptotic *Bcl2* and *BclXl* indicate that both of the examined factors participate in the induction of the apoptotic process, which was also confirmed by the flow cytometer analysis. Similar results were obtained by Min et al. [27], who conducted their research on non-small cell lung cancer H1299 cells that had been treated with caffeic acid. This effect has also been demonstrated on A549 human lung cancer cells and 4T1 breast cancer cells for chlorogenic acid [28,29].

The influence on the caspase activity might also indicate the pro-apoptotic effect of the phenolic acids and an SMF. An increase in the caspase activity was observed after melanoma cells had been treated with caffeic and chlorogenic acid together with a moderate-strength SMF compared to the controls. At the mRNA level, the transcriptional activity of caspase 3 was induced by caffeic acid and the simultaneous exposure of cells to an SMF. Its activation is required in all of the apoptotic signal pathways, both intracellular and extracellular. In turn, caspase 9 is mainly involved in the mitochondrial pathway of apoptosis. Small changes in its expression and activity under the influence of both of the above-mentioned factors might indicate a different mechanism of the activation of programmed cell death, e.g., via the activation of the stress-induced caspases. It might also be due to the ability of the polyphenols to directly modulate different stages of the apoptotic process [30]. On the other hand, a decrease in the copy number of the mRNA caspases was observed as a result of chlorogenic acid and the simultaneous action of an SMF. This phenomenon might be related to the post-transcriptional regulation of gene expression and the post-translational modification of proteins [31]. Our results might also suggest that an SMF can induce apoptosis in a caspase-independent pathway.

It should be emphasized that in our experiment the special structure of the magnetic chambers allows us to obtain the uniform distribution of magnetic flux density over the measurement space of a flask. However, it should be also noted that this exposure system may generate a gradient magnetic field, especially near the edges of the system. Hence, the observed effects may result from the influence of a static magnetic field itself or magnetic field gradient. It was reported that gradient magnetic forces may affect paramagnetic free radicals and thus cell fate [32]. Furthermore, Zablotskii et al. [33] showed that the exposure to a high-gradient magnetic field caused a change in cytoskeleton organization, DNA organization and thus in gene expression of mesenchymal stem cells. It was also proved that a high magnetic field has an effect on the diffusion of diamagnetic and paramagnetic molecules in cell cytoplasm, which may modify cell signaling pathways and cellular processes [32]. Also, a moderate-strength magnetic field may interact with lipids, proteins and glycans of plasma membranes, changing their structures and triggering a cascade of cell responses [34]. In turn, Panczyk and Camp [35] suggested that Lorentz forces do not affect ions or larger molecules in solutions and therefore an SMF has no effect on the structure of molecules. In fact, the observed effect of an SMF and phenolic acids applied together is very difficult to explain at the molecular level and requires further detailed research.

It should be also highlighted that an SMF inhibits the growth of cancer cells while having a minimal effect on normal cells. Moreover, the synergistic effect of an SMF with anticancer drugs was observed [36]. Similar results were also found by Abusoglu and Ozturk [37], who suggested a synergistic effect of an SMF with flavonoids such as quercetin and hesperetin on breast cancer cells. They concluded that an MF might support the anticancer properties of natural compounds via mitochondria-related apoptosis pathway.

In conclusion, our study indicated that both phenolic acids can be used supportively in the treatment of melanoma. The use of natural compounds and a physical agent can be beneficial in reducing the side effects of the current therapeutic strategies. Importantly, caffeic acid and its derivatives have been shown to promote the radio- and chemo-sensitivity of cancer cells [38]. Our results suggest that caffeic acid is more pro-apoptotic than chlorogenic acid, and this remains the case when used in combination with a moderate-strength SMF. However, in combination with an SMF, this effect was weaker at the mRNA and caspase activity levels than when the cells were cultured only in the presence of the polyphenols. Nevertheless, further research is needed to elucidate the interactions between an SMF and the polyphenols and their effects on other melanoma cells, as well as on a normal melanocyte cell line at the molecular level.

## 4. Materials and Methods

The studies were performed on the C32 cell line (amelanotic melanoma, ATCC, CRL-1585; Manassas, VA, USA).

### 4.1. Cell Culture Conditions

The C32 cells were maintained in Dulbecco’s Modified Eagle Medium (DMEM, Lonza, Basel, Switzerland) that had been supplemented with 10% FBS (fetal bovine serum), amphotericin B (0.25 µg/mL) and penicillin (100 U/mL) in a 5% CO_2_ incubator (Heraeus, Hanau, Germany) at 37 °C. The number and viability of the cells were assessed in a Countess^TM^ Automated Cell Counter (Invitrogen, Carlsbad, CA, USA) after being stained with 0.4% trypan blue staining. Cells between passages four and eight were used for this experiment.

For the experiment, the C32 cells were cultured in 25 cm^2^ cell culture flasks at a density of 2 × 10^6^ cells (Sarstedt, Nümbrecht, Germany). After 24 h, the cells were treated with chlorogenic acid (CGA, IUPAC name: (1*S*,3*R*,4*R*,5*R*)-3-{[(2*E*)-3-(3,4-Dihydroxyphenyl)prop-2-enoyl]oxy}-1,4,5-trihydroxycyclohexane-1-carboxylic acid, Sigma-Aldrich, St. Louis, MO, USA) or caffeic acid (CA, IUPAC name: (2E)-3-(3,4-dihydroxyphenyl)prop-2-enoic acid, Sigma-Aldrich, St. Louis, MO, USA) at a concentration of 1 mmol/L (Figure S3) and then exposed to a moderate-intensity SMF (0.7 T) that was generated by permanent magnets in special magnetic chambers that are designed for studying the impact of an SMF in vitro. These chambers consist of a ferromagnetic yoke, which constitute the bottom and cover of the chambers, and permanent magnets. They are enclosed by lateral, front and back walls. The front wall is fitted with a window with dimensions that correspond to the lateral dimensions of a culture flask. Such a construction of test chambers permits the uniform distribution of magnetic flux density over the measurement space of a flask [39,40]. At the same time, the control cells were cultured in a chamber, in which steel was used instead of a magnet (0.0 T). The CGA and CA stock solutions were prepared in phosphate buffered saline (PBS, Sigma-Aldrich, St. Louis, MO, USA) and then diluted in the culture medium. The concentration of both acids was selected based on previous cytotoxicity studies, which had shown that a concentration of 1 mmol/L only reduced the viability of the C32 cells and had no significant effect on the viability of normal human dermal fibroblasts [25,26]. Moreover, the biological effects of a moderate-strength SMF do not exhibit cytotoxic properties as was indicated in previous reports [41].

The CA- or CGA-treated cells were maintained in the test chambers at 37 °C in a 5% CO_2_ incubator for 24 h.

### 4.2. Apoptosis Assay

The apoptosis of the C32 cells that had been treated with chlorogenic or caffeic acid and simultaneously exposed to an SMF was determined using flow cytometry and an eBioscience™ Annexin V Apoptosis Detection Kit FITC (Invitrogen, Carlsbad, CA, USA).

For this purpose, the cells were harvested using an Accutase Solution (Sigma-Aldrich, St. Louis, MO, USA), then washed with PBS and resuspended in the 1× Binding Buffer. In the next step, the cells were stained with fluorochrome-conjugated Annexin V and Propidium Iodide according to the manufacturer’s instructions. Apoptosis was detected using an Attune^TM^ NxT Flow Cytometer (Thermo Fisher Scientific, Waltham, MA, USA).

### 4.3. RNA Extraction

Total RNA was extracted from the cells intended for molecular analysis using a TRIzol reagent (Invitrogen, Carlsbad, CA, USA) according to the manufacturer’s instructions. The quality and the quantity of the RNA extracts were determined electrophoretically and spectrophotometrically as was previously reported [25].

### 4.4. Real Time RT-qPCR Assay

The RT-qPCR technique was used to determine the mRNA level of the genes that are associated with apoptosis: *BAX* (Bcl-2-associated X protein), *Bcl2* (B-cell lymphoma 2), *BclXl* (B-cell lymphoma-extra-large), *Casp3* (caspase 3) and *Casp9* (caspase 9). The gene expression was evaluated using SYBR Green I chemistry (SensiFAST SYBR No-ROX One-Step, Bioline, London, UK) and a LightCycler^®^ 480 Instrument II (Roche Life Science, Basel, Switzerland). The oligonucleotide primers were commercially available (Sigma-Aldrich, St. Louis, MO, USA) (Table S1).

A melting curve analysis was also performed to confirm the specificity of the amplification and the absence of any primer dimers. The 2^−(ΔCt)^ method with *β-actin* as the reference gene (where the ΔCt = Ct of our gene of interest–Ct of *β-actin*) was used to assess the relative mRNA expression of the apoptotic genes [42].

### 4.5. Western Blot Analysis

Cell lysis was performed using a PathScan cell lysis buffer (Cell Signaling, Danvers, MA, USA) that had been supplemented with phenylmethylsulfonyl fluoride and protease inhibitors (Roche, Burgess Hill, UK). After cell lysis, the total protein concentration was measured using a bicinchoninic acid (BCA) assay. Equal amounts of the protein were loaded and separated using SDS-PAGE, and then transferred onto a low autofluorescence immobilon-FL PVDF membrane (Millipore, Burlington, MA, USA). The membranes were incubated with primary rabbit anti-caspase 3, mouse anti-caspase 9 (1:1000) (Cell Signaling), rabbit anti-Bcl-x (0.3 μg/mL) (R&D Systems) and mouse anti-β-actin (1:5000) (Sigma-Aldrich) antibodies at 4 °C overnight, followed by incubation with goat anti-rabbit that had been conjugated with fluorescent dye IRDye800 and goat anti-mouse that had been conjugated with fluorescent dye IRDye700 secondary antibodies (LI-COR Biosciences, Lincoln, NE, USA). The proteins of interest were visualized using a LI-COR Odyssey Infrared Imaging System.

### 4.6. Caspase Activity Assay

The caspase 3 and 9 activity was determined in the cell lysates using a commercially available Caspase-3 Assay Kit (Colorimetric) and a Caspase-9 Assay Kit (Colorimetric) (Abcam, Cambridge, UK) according to the manufacturer’s instructions. The absorbance at wavelengths of 405 nm was read on a UV/Visible Spectrophotometer SP-8001 (Metertech, Taiwan). The caspase activity was calculated relative to the cellular protein content, which was measured using a Pierce™ BCA Protein Assay Kit (Thermo Scientific, Waltham, MA, USA).

### 4.7. DNA/RNA Oxidative Damage Marker Concentration

The oxidative damage marker concentration (8-hydroxy-2′-deoxyguanosine) was measured using a DNA/RNA Oxidative Damage (High Sensitivity) ELISA Kit (Cayman Chemical, Ann Arbor, MI, USA) in the cell culture medium according to the manufacturer’s instructions. The absorbance at a wavelength of 405 nm was read on a Wallac 1420 VICTOR microplate reader (PerkinElmer, Waltham, MA, USA).

### 4.8. Determining ROS Production

Intracellular ROS production was estimated using flow cytometry (AttuneTM NxT Flow Cytometer, Thermo Fisher Scientific, Waltham, MA, USA) using 2′,7′-dichlorodihydrofluorescein diacetate (H_2_DCF-DA; Thermo Fisher Scientific, Waltham, MA, USA) according to the manufacturer’s protocol. The fluorescence intensity of the DCF represented the quantity of intracellular ROS. H_2_O_2_ (0.1 mmol/L) was used as the positive control.

### 4.9. Statistical Analyses

The statistical analyses were performed using Statistica 13.3 software (StatSoft, Tulsa, OK, USA) and the level of significance was set at *p* < 0.05. In the statistical analyses, the one-way ANOVA and Tukey post hoc tests were used due to the normal distribution of the data.

To analyze the interactions between the physical and chemical factors, we used the coefficient of drug interaction (CDI), where a CDI value <1, =1 or >1 indicates that the tested factors are synergistic, additive or antagonistic, respectively [43].

The samples were tested in triplicate for all the assays.

## Data Availability

Not applicable.

## Supplementary Materials

The following supporting information can be downloaded at: https://www.mdpi.com/article/10.3390/ijms23073859/s1.

## References

[1] Kiraz Y., Adan A., Kartal Yandim M., Baran Y. (2016). Major apoptotic mechanisms and genes involved in apoptosis. Tumour Biol..

[2] Rusin P., Jabłońska K. (2020). Disturbances in the Mechanism of Apoptosis as One of the Causes of the Development of Cancer Diseases. Studia Ecol. Et Bioethicae.

[3] Soengas M.S., Lowe S.W. (2003). Apoptosis and melanoma chemoresistance. Oncogene.

[4] Pfeffer C.M., Singh A.T.K. (2018). Apoptosis: A Target for Anticancer Therapy. Int. J. Mol. Sci..

[5] Tsao R. (2010). Chemistry and Biochemistry of Dietary Polyphenols. Nutrients.

[6] Williamson G. (2017). The role of polyphenols in modern nutrition. Nutr. Bull..

[7] Yamanaka N., Oda O., Nagao S. (1997). Prooxidant activity of caffeic acid, dietary non-flavonoid phenolic acid, on Cu^2+^-induced low density lipoprotein oxidation. FEBS Lett..

[8] Maurya D.K., Devasagayam T.P. (2010). Antioxidant and prooxidant nature of hydroxycinnamic acid derivatives ferulic and caffeic acids. Food Chem. Toxicol..

[9] Zhang L., Wang J., Wang H., Wang W., Li Z., Liu J., Yang X., Ji X., Luo Y., Hu C. (2016). Moderate and strong static magnetic fields directly affect EGFR kinase domain orientation to inhibit cancer cell proliferation. Oncotarget.

[10] Gellrich D., Schmidtmayer U., Eckrich J., Hagemann J., Becker S., Strieth S. (2018). Modulation of Exposure to Static Magnetic Field Affects Targeted Therapy of Solid Tumors In Vivo. Anticancer Res..

[11] Zafari J., Vazini H., Jouni F.J., Abdolmaleki P., Monajemi R., Shams E., Satari M. (2018). Anticancer Effects of Moderate Static Magnetic Field on Cancer Cells In Vitro. Res. Mol. Med..

[12] Chen W.T., Lin G.B., Lin S.H., Lu C.H., Hsieh C.H., Ma B.L., Chao C.Y. (2018). Static magnetic field enhances the anticancer efficacy of capsaicin on HepG2 cells via capsaicin receptor TRPV1. PLoS ONE.

[13] Abdal Dayem A., Choi H.Y., Yang G.M., Kim K., Saha S.K., Cho S.G. (2016). The Anti-Cancer Effect of Polyphenols against Breast Cancer and Cancer Stem Cells: Molecular Mechanisms. Nutrients.

[14] Briguglio G., Costa C., Pollicino M., Giambò F., Catania S., Fenga C. (2020). Polyphenols in cancer prevention: New insights (Review). Int. J. Funct. Nutr..

[15] Prakash M.D., Stojanovska L., Feehan J., Nurgali K., Donald E.L., Plebanski M., Flavel M., Kitchen B., Apostolopoulos V. (2021). Anti-cancer effects of polyphenol-rich sugarcane extract. PLoS ONE.

[16] Villota H., Moreno-Ceballos M., Santa-González G.A., Uribe D., Castañeda I.C.H., Preciado L.M., Pedroza-Díaz J. (2021). Biological Impact of Phenolic Compounds from Coffee on Colorectal Cancer. Pharmaceuticals.

[17] Montenegro J., Dos Santos L.S., de Souza R., Lima L., Mattos D.S., Viana B., da Fonseca Bastos A., Muzzi L., Conte-Júnior C.A., Gimba E. (2021). Bioactive compounds, antioxidant activity and antiproliferative effects in prostate cancer cells of green and roasted coffee extracts obtained by microwave-assisted extraction (MAE). Food Res. Int..

[18] Feriotto G., Tagliati F., Giriolo R., Casciano F., Tabolacci C., Beninati S., Khan M.T.H., Mischiati C. (2021). Caffeic Acid Enhances the Anti-Leukemic Effect of Imatinib on Chronic Myeloid Leukemia Cells and Triggers Apoptosis in Cells Sensitive and Resistant to Imatinib. Int. J. Mol. Sci..

[19] Yang G., Fu Y., Malakhova M., Kurinov I., Zhu F., Yao K., Li H., Chen H., Li W., Lim D.Y. (2014). Caffeic acid directly targets ERK1/2 to attenuate solar UV-induced skin carcinogenesis. Cancer Prev. Res..

[20] Pelinson L.P., Assmann C.E., Palma T.V., da Cruz I., Pillat M.M., Mânica A., Stefanello N., Weis G., de Oliveira Alves A., de Andrade C.M. (2019). Antiproliferative and apoptotic effects of caffeic acid on SK-Mel-28 human melanoma cancer cells. Mol. Biol. Rep..

[21] Jin S., Kim K.C., Kim J.S., Jang K.I., Hyun T.K. (2020). Anti-Melanoma Activities and Phytochemical Compositions of Sorbus commixta Fruit Extracts. Plants.

[22] Pop T.D., Diaconeasa Z. (2021). Recent Advances in Phenolic Metabolites and Skin Cancer. Int. J. Mol. Sci..

[23] Martino R., Barreiro Arcos M.L., Peralta I., Marrassini C., Saint Martin E.M., Cogoi L., Cremaschi G., Alonso M.R., Anesini C. (2021). Antiproliferative activity of aqueous and polyphenol-rich extracts of Larrea divaricata Cav. on a melanoma cell line. Nat. Prod. Res..

[24] Huang L., Chen J., Quan J., Xiang D. (2021). Rosmarinic acid inhibits proliferation and migration, promotes apoptosis and enhances cisplatin sensitivity of melanoma cells through inhibiting ADAM17/EGFR/AKT/GSK3β axis. Bioengineered.

[25] Kimsa-Dudek M., Krawczyk A., Synowiec-Wojtarowicz A., Dudek S., Pawłowska-Góral K. (2020). The impact of the co-exposure of melanoma cells to chlorogenic acid and a moderate-strength static magnetic field. J. Food Biochem..

[26] Kimsa-Dudek M., Krawczyk A., Synowiec-Wojtarowicz A., Biały Ł., Młynarczuk-Biały I. (2020). Redox homeostasis in melanoma cells treated with caffeic acid. Advances in Biomedical Research—from COVID to Medical Humanities.

[27] Min J., Shen H., Xi W., Wang Q., Yin L., Zhang Y., Yu Y., Yang Q., Wang Z.N. (2018). Anticancer Activity of Combined Use of Caffeic Acid with Paclitaxel Enhances Apoptosis of Non-Small-Cell Lung Cancer H1299 Cells in Vivo and in Vitro. Cell Physiol. Biochem..

[28] Yamagata K., Izawa Y., Onodera D., Tagami M. (2018). Chlorogenic acid regulates apoptosis and stem cell marker-related gene expression in A549 human lung cancer cells. Mol. Cell. Biochem..

[29] Changizi Z., Moslehi A., Rohani A.H., Eidi A. (2020). Chlorogenic acid inhibits growth of 4T1 breast cancer cells through involvement in Bax/Bcl2 pathway. J. Cancer Res. Ther..

[30] D’Archivio M., Santangelo C., Scazzocchio B., Varì R., Filesi C., Masella R., Giovannini C. (2008). Modulatory Effects of Polyphenols on Apoptosis Induction: Relevance for Cancer Prevention. Int. J. Mol. Sci..

[31] Vogel C., Marcotte E.M. (2012). Insights into the regulation of protein abundance from proteomic and transcriptomic analyses. Nat. Rev. Genet..

[32] Zablotskii V., Polyakova T., Dejneka A. (2022). Effects of High Magnetic Fields on the Diffusion of Biologically Active Molecules. Cells.

[33] Zablotskii V., Lunov O., Novotna B., Churpita O., Trošan P., Holá V., Sykova E., Dejneka A., Kubinová Š. (2014). Down-regulation of adipogenesis of mesenchymal stem cells by oscillating high-gradient magnetic fields and mechanical vibration. Appl. Phys. Lett..

[34] Vergallo C., Panzarini E., Tenuzzo B.A., Mariano S., Tata A.M., Dini L. (2020). Moderate Static Magnetic Field (6 mT)-Induced Lipid Rafts Rearrangement Increases Silver NPs Uptake in Human Lymphocytes. Molecules.

[35] Panczyk T., Camp P.J. (2021). Lorentz forces induced by a static magnetic field have negligible effects on results from classical molecular dynamics simulations of aqueous solutions. J. Mol. Liq..

[36] Zhang X., Yarema K., Xu A. (2017). Biological Effects of Static Magnetic Fields.

[37] Abusoglu G., Ozturk B. (2020). Effect of static magnetic field with quercetin and hesperetin on MCF-7 and MDA MB-231 breast cancer cells. Turk. J. Biochem..

[38] Mirzaei S., Gholami M.H., Zabolian A., Saleki H., Farahani M.V., Hamzehlou S., Far F.B., Sharifzadeh S.O., Samarghandian S., Khan H. (2021). Caffeic acid and its derivatives as potential modulators of oncogenic molecular pathways: New hope in the fight against cancer. Pharmacol. Res..

[39] Gawron S., Glinka M., Wolnik T. (2012). Magnetyczna komora badawcza dedykowana do hodowli komórek. Zesz. Probl. Masz. Elektr..

[40] Glinka M., Gawron S., Sieroń A., Pawłowska-Góral K., Cieślar G., Sieroń-Stołtny K. (2013). Test chambers for cell culture in static magnetic field. J. Magn. Mater..

[41] Dini L., Abbro L. (2005). Bioeffects of moderate-intensity static magnetic fields on cell cultures. Micron.

[42] Schmittgen T.D., Livak K.J. (2008). Analyzing real-time PCR data by the comparative C(T) method. Nat. Protoc..

[43] Soica C., Oprean C., Borcan F., Danciu C., Trandafirescu C., Coricovac D., Crăiniceanu Z., Dehelean C.A., Munteanu M. (2014). The Synergistic Biologic Activity of Oleanolic and Ursolic Acids in Complex with Hydroxypropyl-γ-Cyclodextrin. Molecules.

