# Supplementary material for: The Apoptotic Effect of Caffeic or Chlorogenic Acid on the C32 Cells That Have Simultaneously Been Exposed to a Static Magnetic Field
Source: Int J Mol Sci. 2022 Mar 31;23(7):3859. doi: 10.3390/ijms23073859 (PMC8999068; doi:10.3390/ijms23073859)
Supplement: Supplementary file 1 [file ijms-23-03859-s001.zip › Kimsa-Dudek_Suppl.Figure S1.pdf]

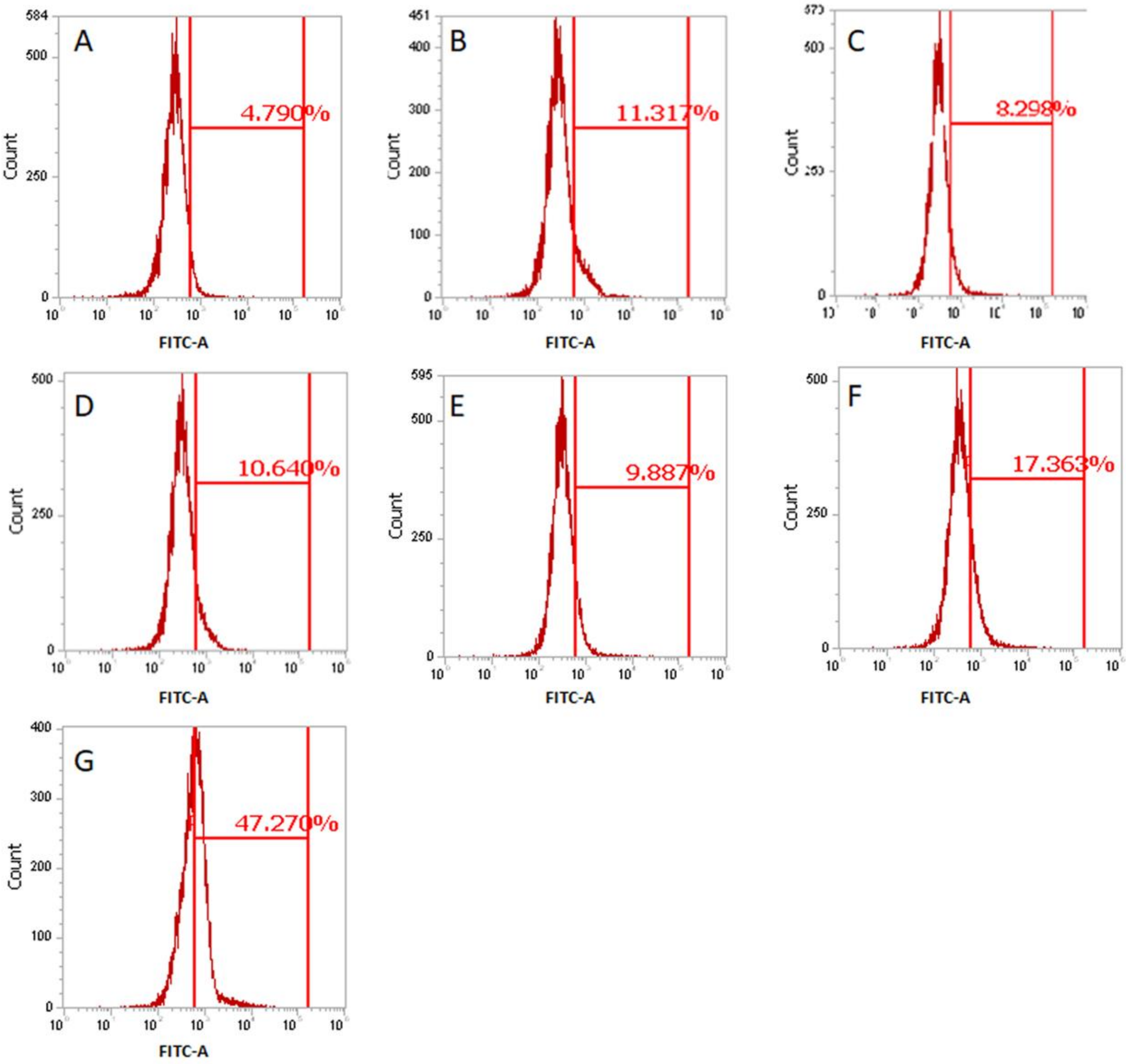

Suppl. Figure S1. Intracellular ROS level in C32 cells (A - control cultures; B - caffeic acid-treated C32 cells; C - chlorogenic acid-treated C32 cells; D - cells that had simultaneously been exposed to caffeic acid and a static magnetic field; E - cells that had simultaneously been exposed to chlorogenic acid and a static magnetic field; F - cells that had been exposed to a static magnetic field; G – H_2O_2-treated cells)
