# Supplementary material for: The Apoptotic Effect of Caffeic or Chlorogenic Acid on the C32 Cells That Have Simultaneously Been Exposed to a Static Magnetic Field
Source: Int J Mol Sci. 2022 Mar 31;23(7):3859. doi: 10.3390/ijms23073859 (PMC8999068; doi:10.3390/ijms23073859)
Supplement: Supplementary file 1 [file ijms-23-03859-s001.zip › Kimsa-Dudek_Suppl.Table S1.pdf]

Suppl. Table S1. Characteristics of the primers that were used for real-time RT-qPCR

Gene	Accession number	Sequence of primers	Tm of primers (˚C)	Tm of PCR amplicon (˚C)	Thermal profile of one-step RT-PCR
Bax	NM_004324	F: 5′- AACTGGACAGTAACATGGAG-3′	56.6	88.4	reverse transcription at 45°C for 10 min<br><br>activation at 95°C for 2 min<br><br>40 cycles:<br>95°C for 5 s<br>60°C for 10 s<br>72°C for 5 s
		R: 5′- TTGCTGGCAAAGTAGAAAAG-3′	59.7		
Bcl2	NM_000633	F: 5′- GATTGTGGCCTTCTTTGAG-3′	59.8	87.1	
		R: 5′- GTTCCACAAAGGCATCC-3′	59.0		
BclXl	NM_001191	F: 5'- ATCTCTTTCTCTCCCTTCAG -3′	59.8	81.7	
		R: 5'- TCTTTCTGGGAAAGCTTGTAG -3'	58.7		
Casp3	NM_004346	F: 5'- AAAGCACTGGAATGACATC -3'	57.6	80.7	
		R: 5'- CGCATCAATTCCACAATTTC -3'	62.8		
Casp9	NM_001229	F: 5′- CTCTACTTTCCCAGGTTTTG-3′	57.9	81.0	
		R: 5′- TTTCACCGAAACAGCATTAG -3′	60.3		
β-actin	NM_001101	F: 5′- GACGACATGGAGAAAATCTG-3′	59.7	88.2	
		R: 5′- ATGATCTGGGTCATCTTCTC-3′	58.0		

F – forward; R – reverse; Tm – melting temperature
