# Supplementary figures and images for: The Apoptotic Effect of Caffeic or Chlorogenic Acid on the C32 Cells That Have Simultaneously Been Exposed to a Static Magnetic Field
Source: Int J Mol Sci. 2022 Mar 31;23(7):3859. doi: 10.3390/ijms23073859 (PMC8999068; doi:10.3390/ijms23073859)

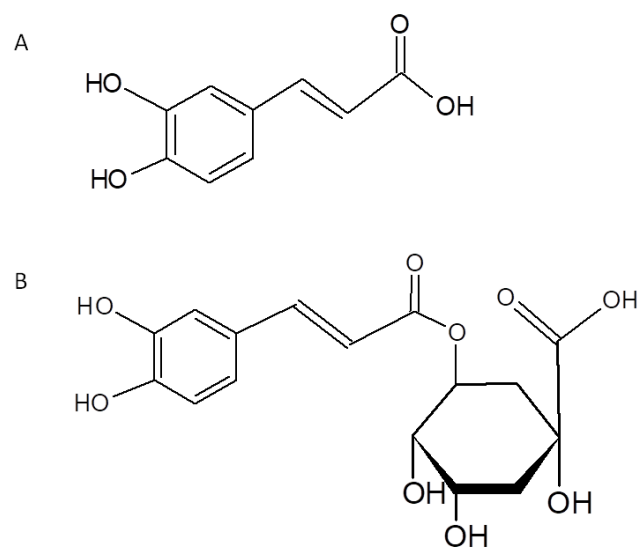

**Suppl. Figure S3.** Chemical structure of caffeic (A) and chlorogenic acid (B)

Supplement: Supplementary file 1 [file ijms-23-03859-s001.zip › Kimsa-Dudek_Suppl.Figure S3.pdf]
